# The role of perspective-taking in suppressing stereotypes about mathematics

**DOI:** 10.1186/s13104-023-06460-6

**Published:** 2023-08-29

**Authors:** Mana Yamamoto, Takashi Oka

**Affiliations:** 1 College of Commerce, Nihon University, Tokyo, Japan; 2 College of Humanities and Sciences, Nihon University, Tokyo, Japan

**Keywords:** Stereotype suppression, Paradoxical effects, Replacement thoughts, Perspective-taking

## Abstract

**Objective:**

When people attempt to suppress stereotypes, they often end up making stereotypical judgments. The adverse effects of this form of suppression are called “paradoxical effects.” This study examined the effect of perspective-taking as a strategy to reduce the paradoxical effects related to stereotype suppression. Specifically, this study addressed stereotypes within the context of women’s mathematical abilities, with Japanese university students as participants. It was predicted that when participants suppressed the stereotype of a woman, those who engaged in perspective-taking toward that woman would make less stereotypical judgments of other women, compared with those who did not. Moreover, as this study focuses on gender stereotypes, an exploratory analysis was conducted to investigate whether the effects of engaging in perspective-taking about women vary depending on the participants’ gender.

**Results:**

Although no significant effect was observed and the hypothesis was not supported, and while the results of this study were statistically inadequate, they suggest that among the female participants, those who did not engage in perspective-taking showed the paradoxical effects of stereotype suppression. However, those paradoxical effects were not observed among those who performed perspective-taking.

## Introduction

When people suppress stereotypes, their stereotypical thinking is often activated, making them more likely to make stereotypical judgments. The adverse effects of this suppression are called paradoxical effects (e.g., [1]; as a review, [2]). For instance, participants who were instructed to suppress stereotypic thoughts produced more stereotypic descriptions and altered their behavior toward members of the target social category [1].

Previous studies have shown that handy replacement thoughts (e.g., if a white bear comes to mind, think of a red Volkswagen) may decrease paradoxical effects (e.g., [3]). It has been shown that replacement thoughts that are person-related in content and have high accessibility, and do not require cognitive resources, may be effective in stereotype suppression (e.g., [4]).

We propose that engaging in perspective-taking when people suppress stereotypes may facilitate the use of effective replacement thoughts. Perspective-taking is the process of imagining oneself in another person’s shoes and envisioning the world from their perspective [5]. Previous studies have shown that during perspective-taking, an individual’s self-concept gets activated (e.g., [5]); this activation does not require cognitive resources, as it is an unconscious process [6]. The present study predicted that when people engage in perspective-taking while suppressing stereotypes, their self-concept becomes a highly accessible replacement thought that does not require cognitive resources; this causes paradoxical effects of a lower degree than when they do not engage in perspective-taking.

### Aims and hypotheses

The objective of this study was to examine the effectiveness of perspective-taking as a strategy to decrease the paradoxical effects of stereotype suppression. Galinsky and Moskowitz [7] conducted a comparison of stereotype suppression and perspective-taking conditions; however, they did not examine the effects of perspective-taking during suppression directly. Therefore, the present study examined the role of perspective-taking during stereotype suppression.

This study focused on gender stereotypes, specifically assessing the stereotype that “women can’t do math” (e.g., [8, 9]). Previous research has demonstrated that gender stereotypes can vary across cultures (e.g., [10]). The current study targeted Japanese university students because Japan, as a cultural context, has demonstrated the existence of stereotypes related to female mathematical competence (e.g., [9]). Furthermore, it is noteworthy that Japan ranks low on the Global Gender Gap Report [11].

Therefore, this study examined whether engaging in perspective-taking about a target woman when suppressing stereotypes about the woman’s mathematical ability would decrease the extent of subsequent stereotypical judgments. We hypothesized that the paradoxical effects would be reduced when perspective-taking is performed during stereotype suppression, compared to when it is not performed.^1^Furthermore, as this study addresses stereotypes about women, we also conducted an exploratory examination of whether the effects of engaging in perspective-taking about women differ by gender.

### Main text

#### Methods

**Design.** This study had a 2 (stereotype suppression: suppression vs. non-suppression) × 2 (perspective-taking: taking vs. non-taking) factor between-participant design. The dependent variables were ratings of a woman’s mathematic ability and estimate of her mathematics test score.

**Participants.** The participants included 329 Japanese university students. However, one participant who did not follow the task instructions for the manipulation of the independent variables was excluded from the analysis. Therefore, 328 participants were finally included in the analysis (196 males, 126 females, and 6 did not disclose their gender; *M*_age_ = 19.20, *SD* = 1.30).

This study was approved by the Research Ethics Committee of the College of Humanities and Sciences, Nihon University (Approval Number: 02–53); it was conducted in 2021 and 2022. Informed consent was obtained from all participants. All methods were performed in accordance with the relevant guidelines and regulations.

**Procedure.** Participants completed an online questionnaire and were randomly assigned to one of four conditions: (2 [stereotype suppression: suppression vs. non-suppression] × 2 [perspective-taking: taking vs. non-taking]). Following Macrae et al. [1], the task of this experiment included presenting a photograph of the target woman to the participants and asking the participant to respond to items regarding the woman’s mathematical abilities. First, the participants completed a task for manipulating the independent variables. In this task, the participants were presented with a photograph of a woman, asked to imagine this person taking a math class, and then asked to freely describe what they imagined. For the stereotype suppression condition, participants were asked to suppress any stereotypes about mathematical ability, whereas no such instruction was given for the non-suppression condition. For the perspective-taking condition, participants were asked to engage in perspective-taking about the person in the photo—to put themselves in their shoes—whereas no such instruction was given for the non-perspective-taking condition. Participants then answered questions to a manipulation check. They responded to one item for the manipulation check regarding stereotype suppression (“When responding about the person in the photo, did you make an effort to avoid thinking in terms of prejudice?”), and to two items for a manipulation check of perspective-taking (“Did you imagine how you would feel if you were in that position?” and “Did you try to put yourself in that person’s shoes?”). Participants responded to the questions using a 7-point scale.

Next, the participants completed a task used to measure the dependent variables. They were presented with a picture of a different woman from the previous task and asked to rate the degree to which she “appears to be unable to do mathematical calculations,” “appears to be bad at mental arithmetic,” and “appears to be weak with numbers,” using a 7-point scale. Participants were also asked to estimate the woman’s math test score on a scale from 0 to 100. Finally, they were asked to indicate their age and gender.

## Results

Table 1 shows the means and standard deviations of the variables and correlations among the variables. The rating score of the woman’s mathematical ability included the mean of the three items: “appear to be unable to do mathematical calculations,” “appears to be bad at mental arithmetic,” and “appears to be weak with numbers” (Cronbach’s *α* = .833).

**Table 1:** Means, standard deviations, and correlations among the variables

	*M*	*SD*	1	2
1 Rating score of mathematical ability	3.95	1.40	—	
2 Estimated score of mathematics test	63.12	17.36	− .649**	—
3 Gender (male: 1, female: 2)			.047	− .090

**p < .01.

First, to confirm that the stereotype suppression and perspective-taking instructions were effective, we conducted Welch’s *t*-test on the manipulation check score for stereotype suppression. The score was significantly higher in the stereotype suppression condition (*M* = 5.14, *SD* = 1.56) than in the non-suppression condition (*M* = 3.84, *SD* = 1.94, *t* (311.17) = 6.68, *p* < .001, *d* = 0.74). The manipulation check score for perspective-taking was the mean for the two items (*r* = .536, *p* < .001). The score for the perspective-taking condition (*M* = 5.15, *SD* = 1.37) was significantly higher than that for the non-perspective-taking condition (*M* = 4.19, *SD* = 1.59, *t* (323.62) = 5.89, *p* < .001, *d* = 0.65). These results indicate that both the stereotype suppression and perspective-taking manipulations were successful.

Next, we examined the effect of perspective-taking on stereotype suppression by conducting a 2 (stereotype suppression: suppression vs. non-suppression) × 2 (perspective-taking: taking vs. non-taking) factor between-participant analysis of variance (two-way ANOVA) with the rating score of the woman’s mathematical ability as the dependent variable. The results showed no significant effects. We also conducted a similar ANOVA with estimated score of the woman’s mathematics test score as the dependent variable, but found no significant effect.

To determine whether the effect of perspective-taking differed by gender, we added gender as a factor and conducted a 2 (stereotype suppression: suppression vs. non-suppression) × 2 (perspective-taking: taking vs. non-taking) × 2 (participant gender: male vs. female) factor between-participant analysis of variance (three-way ANOVA). Analysis of the rating score of the woman’s mathematical ability as the dependent variable showed no significant effects. As the results of the analysis with estimated score of the woman’s mathematics test score as the dependent variable showed a significant interaction effect among the three factors (Table 2, F (1, 314) = 4.15, *p* = .043, *η*^2^_*p*_ = .013), a simple interaction test of stereotype suppression × perspective-taking was conducted on each female and male participant. As the results showed a significant trend in the interaction effect for stereotype suppression × perspective-taking among females (*F* (1, 314) = 2.91, *p* = .089, *η*^2^_p_ = .023), a simple-simple main effect test revealed that in the non-taking condition for females, the score was lower in the stereotype suppression condition than in the non-suppression condition (*F* (1, 314) = 4.09, *p* = .044, *η*^2^_p_ = .058). Among males, the simple interaction effect of stereotype suppression × perspective-taking was not significant.

**Table 2:** Means and standard deviations for the estimated score of mathematics test in each condition

Male Participants					Female Participants			
Perspective-taking condition			Non-taking condition		Perspective-taking condition		Non-taking condition	
	Stereotype suppression condition (*n* = 48)	Non-suppression condition (*n* = 50)	Stereotype suppression condition (*n* = 49)	Non-suppression condition (*n* = 49)	Stereotype suppression condition (*n* = 28)	Non-suppression condition (*n* = 30)	Stereotype suppression condition (*n* = 37)	Non-suppression condition (*n* = 31)
*M*	64.06	66.00	65.90	62.27	62.46	60.40	57.43	65.97
*SD*	19.43	19.28	18.03	16.71	15.93	14.39	17.04	14.22

## Discussion

This study examined whether perspective-taking reduces paradoxical effects when suppressing stereotypes. The results of our analysis showed no significant effect, and the hypothesis was not supported. There are two possible reasons why the hypothesis was not supported in this study. First, explicit instructions were provided for stereotype suppression and measurement. Specifically, it is conceivable that the effects of stereotype suppression manipulation could have persisted in the subsequent stereotype measurement task. In the future, it will be necessary to enhance the segregation between the suppression task and subsequent stereotype measurement task through the incorporation of filler tasks and refined instructions. Moreover, it is plausible that the measurement of stereotype-based judgments could have been subject to an avoidance of negative responses due to social desirability concerns. Moving forward, it is imperative to incorporate measurement tasks for stereotype-based judgments that minimize the impact of social desirability. Indeed, previous research has shown that explicit and implicit gender stereotypes have been shown to make different predictions regarding performance related to mathematics (e.g., [14, 15]). Therefore, in the future, it becomes essential to utilize assessment tasks such as lexical decision tasks or the Implicit Association Test (IAT) that are less susceptible to the influence of participants’ intentions and consciousness. Furthermore, a comparative analysis between explicit and implicit responses would be necessary, facilitating a more comprehensive understanding by delving into latent indicators less influenced by participants’ awareness and intentions. Second, this study presented facial photographs of the target individuals using online forms. Compared to face-to-face contact, it is possible that the activation of stereotypes and engagement in perspective-taking were less likely to occur.

As the present study addressed stereotypes about women, we also conducted an exploratory analysis of whether the effects of perspective-taking about women differed by gender. The results did show a significant trend; the estimated math test scores of the female participants who did not perform perspective-taking were lower when they engaged in stereotype suppression than when they did not. This result is consistent with the previous finding that stereotype suppression leads to paradoxical effects (e.g., [1]). However, among the female participants who engaged in perspective-taking, there was no significant difference between the stereotype suppression and non-suppression conditions. These results suggest that, for women, perspective-taking may reduce the paradoxical effects of suppressing stereotypes about women’s mathematical abilities. Although the results of this study showed a significant trend, distinct findings emerged for male and female participants. Future studies should examine how the role of perspective-taking differs depending on the relationship between the target group and the perspective taker.

### Limitations

This study has two notable limitations. First, it proposed that replacement thoughts would arise from the self-concept activated by perspective-taking; however, our results do not specify the content of those replacement thoughts. In future studies, the content of the replacement thoughts actually used should be examined. Second, this study presented outcomes exclusively derived from participants in Japan. Given the potential for stereotypes to exhibit cultural variations (e.g., [10]), it is crucial to account for cultural factors when interpreting and extending the implications of the results. In the future, it is necessary to conduct investigations that also consider cultural factors.

## Data Availability

The data that support the findings of this study are available from the corresponding author upon reasonable request.

## Footnotes

1 In this study, self-esteem was measured as an individual difference in self-concept, using the 10-item Japanese version of Rosenberg’s [12] Self-Esteem Scale [13]. Analysis of covariance was performed with the self-esteem scores set as a covariate, but the results were similar to those of this study, which did not set a covariate.

## Abbreviations

ANOVA: Analysis of variance
IAT: Implicit Association Test
